# Enhanced In Vitro Refolding of Fibroblast Growth Factor 15 with the Assistance of SUMO Fusion Partner

**DOI:** 10.1371/journal.pone.0020307

**Published:** 2011-05-31

**Authors:** Bo Kong, Grace L. Guo

**Affiliations:** Department of Pharmacology, Toxicology, and Therapeutics, University of Kansas Medical Center, Kansas City, Kansas, United States of America; St. Georges University of London, United Kingdom

## Abstract

Fibroblast growth factor 15 (Fgf15) is the mouse orthologue of human FGF19. Fgf15 is highly expressed in the ileum and functions as an endocrine signal to regulate liver function, including bile acid synthesis, hepatocyte proliferation and insulin sensitivity. In order to fully understand the function of Fgf15, methods are needed to produce pure Fgf15 protein in the prokaryotic system. However, when expressed in *Escherichia coli (E. coli)*, the recombinant Fgf15 protein was insoluble and found only in inclusion bodies. In the current study, we report a method to produce recombinant Fgf15 protein in *E. coli* through the use of small ubiquitin-related modifier (SUMO) fusion tag. Even though the SUMO has been shown to strongly improve protein solubility and expression levels, our studies suggest that the SUMO does not improve Fgf15 protein solubility. Instead, proper refolding of Fgf15 protein was achieved when Fgf15 was expressed as a partner protein of the fusion tag SUMO, followed by *in vitro* dialysis refolding. After refolding, the N-terminal SUMO tag was cleaved from the recombinant Fgf15 fusion protein by ScUlp1 (Ubiquitin-Like Protein-Specific Protease 1 from *S. cerevisiae*). With or without the SUMO tag, the refolded Fgf15 protein was biologically active, as revealed by its ability to reduce hepatic Cyp7a1 mRNA levels in mice. In addition, recombinant Fgf15 protein suppressed Cyp7a1 mRNA levels in a dose-dependent manner. In summary, we have developed a successful method to express functional Fgf15 protein in prokaryotic cells.

## Introduction

Fibroblast growth factor 15 (Fgf15) is mainly expressed in mouse ileum and colon and is not expressed in liver. Its human homologue is FGF19. Fgf15 contains 218 amino acids (aa), including a predicted 25-aa signal peptide and a 193-aa secreted mature protein [1], and shares 50% aa sequence similarity with FGF19. Fgf15 functions as an enterohepatic hormone in that it is produced in the small intestine, but travels to the liver via portal circulation, and then binds to its receptor, FGFR4 that is expressed in hepatocytes [2]. Activation of FGFR4 leads to activation of downstream signaling pathways and results in suppression of gene transcription of Cyp7a1 gene that encodes cholesterol 7α-hydroxylase, the rate-limiting enzyme for bile acid synthesis in the liver [3], [4]. Therefore, Fgf15/FGF19 plays a critical role in regulating cholesterol and bile acid homeostasis. Over-expression of FGF19 in FGF19-transgenic mice results in liver tumor formation, increased energy expenditure, decreased adiposity, and resistance to weight gain in response to a high fat diet [5], [6]. Similar effects have also been observed in mice treated intravenously with recombinant FGF19 [7], suggesting that Fgf15/FGF19 are important to physiology, in addition to regulating bile acid homeostasis. The study of Fgf15 function in mice can be a valuable research tool because mouse models are widely used to study human physiology and pathology. Previously, Fgf15 protein has been produced by adenoviral infection of mammalian cells [1]. However, in order to produce large quantities of biologically active Fgf15 protein to study its effects *in vivo*, it will be ideal to develop a prokaryotic system to produce biologically active Fgf15 protein.

The *E. coli* system is a popular and well characterized prokaryotic host system for heterologous protein expression. This system produces large quantities of protein in a shorter period of time and at lower cost. However, the high yield of heterologous protein in *E. coli* often leads to improper protein folding, which results in insoluble and non-functional proteins that are aggregated in inclusion bodies [8], [9]. Even though aggregation of recombinant protein in inclusion bodies provides an easy method for protein isolation and purification, refolding of the recombinant protein *in vitro* to gain biological activity often presents great challenges [10].

Extensive efforts have been made to promote the expression of soluble recombinant proteins in *E. coli*. One strategy is to reduce protein synthesis rate by lowering incubation temperature and inducing pressure [11]. Another widely adapted strategy to improve the solubility of recombinant proteins is to add a fusion tag such as glutathione-s-transferase (GST) [12], [13], maltose binding protein (MBP) [14], [15], NusA [16], thioredoxin (Trx) [12], [17], or small ubiquitin-like modifier (SUMO) [18], [19] to the target protein. Fusion tags have been shown to improve protein solubility as well as protein expression level in the *E. coli* system. However, the formation of inclusion bodies in *E. coli* is complicated and the mechanism for this formation is not yet clear. For example, when using *E. coli* as a protein expression system, some eukaryotic proteins are highly likely to aggregate, regardless of the type of fusion tag used to improve protein solubility. This aggregation can lead to cumbersome and challenging procedures for *in-vitro* refolding. When a protein produced in the prokaryotic system is highly insoluble, the only option to make it soluble is to use a low-yield eukaryotic expression system. However, the low-yield protein will make the downstream protein purification more difficult.

SUMO is a ubiquitin-related protein and regulates the activity of a wide variety of cellular target proteins by covalent modification of the target protein's lysine residues [20]. In the last decade, SUMO protein has been successfully developed as a robust prokaryotic protein expression system. Previous researches show that SUMO improves protein expression levels and solubility when it is fused to a protein's N-terminus by inherited chaperone properties, thus making SUMO a useful tag for improving heterologous protein expression in prokaryotic cells [18], [19].

In the current study, SUMO fusion tag was attached to the N-terminus of Fgf15 and the fusion proteins were expressed in *E. coli*. In this system, Fgf15 was still expressed in the form of inclusion bodies, but the Fgf15 protein was properly refolded after dialysis refolding steps with the assistance of fusion moiety SUMO. By using this protocol, we were able to produce and purify biologically active Fgf15 protein in large quantity.

## Materials and Methods

### Ethics Statement

Mice were bred and maintained in the Laboratory of Animal Research facility at the University of Kansas Medical Center. Mice were housed in rooms under a standard 12-hr light/dark cycle with access to chow and water ad libitum. All protocols and procedures were approved by the Laboratory of Animal Research Committee at the University of Kansas Medical Center and are in accordance with the NIH and AALAC Guidelines (protocol# 2007-1699). All experiments were performed with age-matched 10–16 week old male mice.

### Plasmid construction

**Figure 1:**
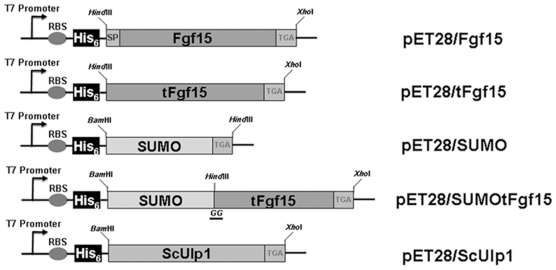
Schematic of expression vectors. pET28a(+) vector backbone has been used to construct the expression vectors. His6-tag has been attached to the N-terminus of the target protein, and the stop codon, TGA, has been added in front of the XhoI restriction enzyme site. Two glycine amino acids have been introduced to the C-terminus of SUMO protein, which is required for the ScUlp1 cleavage. SP, signal peptide.

Yeast (*S. cerevisiae*) SUMO and the C-terminal protease domain of Ulp1 (ScUlp1) were PCR amplified from yeast genomic DNA using Phusion High-Fidelity DNA Polymerase (NEB) by primers: ScUlp1_F: CGCGGGATCCAAACTTGTTCCTGAATTAAATG, ScUlp1_R: GACTCTCGAGTTATTTTAAAGCGTCGGTTAAAATC, SUMO_F: CGCGGATCCATGTCGGACTCAGAAGTCAATC, SUMO_R: CGCAAGCTT *ACCACC*AATCTGTTCTCTGTG. The Fgf15 gene was amplified from mouse intestinal total cDNA using the primers Fgf15_F: CGCAAGCTTATGGCGAGAAAGTGGAACGG and Fgf15_R: GACTCTCGAGTCATTTCTGGAAGCTGGGAC. Truncated Fgf15 (tFgf15) gene without a coding fragment for the signal peptide was amplified using primers tFgf15_F: CGCAAGCTTCGTCCCCTGGCTCAGCAATC, and tFgf15_R: GACTCTCGAGTCATTTCTGGAAGCTGGGAC. Sequences underlined were the restriction enzyme sites used for inserting amplified fragments into the bacterial expression vector, pET28a(+). Two additional glycines [19], [20], [21] required for ScUlp1 protease to cleave the SUMO tag were inserted between the SUMO fusion tag and Fgf15 protein with an N-terminal His6-tag. All plasmids used for protein expression are shown in **Figure 1**.

### Protein expression and purification of inclusion bodies

The BL21 (DE3) *E. coli* strain (Novagen) was transformed with plasmid constructs. A single-colony transformant was inoculated into 5 ml Luria Bertani (LB) medium containing 50 µg/ml kanamycin and grown overnight at 37°C. The culture was transferred the following day to 200 ml fresh LB medium with kanamycin and was allowed to grow at 37°C until the optical density (OD600) reached about 0.6. Isopropylthiogalactoside (IPTG) was then added to a final concentration of 0.3 mM to induce protein expression at 30°C for 4 hrs. The cells were harvested by centrifugation at 8,000 g for 10 mins and resuspended in lysis buffer (50 mM Tris-HCl, pH 8.5, 0.5 mM EDTA and 300 mM NaCl). Lysozyme (0.5 mg/ml, Sigma) and DNA nucleases (5 units/ml, Fermentas) were added to the suspension, and the suspension was left at room temperature for 30 mins to lyse the cells. Ultrasonication was then performed for further cell disruption. After sonication, the suspension was centrifuged at 10,000 g for 30 mins at 4°C. The resulting supernatant representing the soluble protein fraction and the pellet were applied to 12% or 15% SDS-PAGE gels to check the recombinant protein expression and solubility.

Inclusion bodies were separated from the soluble fraction by centrifugation at 8,000 g. Impurities trapped within the inclusion body pellet were removed using a series of detergent and buffer washes. After centrifugation, the pellet was washed twice with lysis buffer containing 2 M Urea and 1% Triton X-100, followed by two more washes with lysis buffer containing 2 M Urea, and samples were stired for 30 mins at each step. After washing, the precipitated inclusion bodies were solubilized with IB solubilization buffer (20 mM Tris-HCl, pH 8.5, 8 M urea, 0.3 M NaCl, 20 mM imidazole). After incubation at room temperature for 2 hrs, the solution was centrifuged at 20,000 g for 15 mins to remove precipitated proteins. The supernatant was processed for protein purification by binding to a Ni-NTA resin, which was pre-equilibrated with IB solubilization buffer. After binding at 4°C overnight, the Ni-NTA resin was collected by low centrifugation at 1,500 g and washed twice with 50 ml IB solublization buffer. Finally, the bound protein was eluted with 20 ml IB elution buffer (IB solubilization buffer containing 200 mM imidazole) before proceeding with the *in-vitro* refolding process.

### In vitro refolding of Fgf15 by dialysis

Protein concentration of the purified inclusion bodies was quantified by Bradford protein assay (Bio-Rad) and adjusted to 1 mg/mL using IB refolding buffer (20 mM Tris-HCl, pH 8.5, 0.3 M NaCl, 1 mM EDTA) with 8 M urea. A final concentration of 5 mM glutathione/0.5 mM oxidized glutathione was added to the solution and gently stirred overnight to reduce the protein. 20 ml of the reduced protein solution was then dialyzed against 1,000 ml IB refolding buffer containing 6, 3 and 2 M urea to gradually remove urea at 4°C over the next 24 hrs. 5 mM DTT was also included in this stage. After removal of urea denaturant, the protein was further dialyzed against IB refolding buffer with a stepwise reducing concentration of DTT from 5, 2 and 1 mM to allow for disulfide bond reshuffling and oxidation in the next 48 hrs. The folded protein was then dialyzed against PBS buffer for 24 hrs, and the PBS buffer was changed three times during this process. Finally, the protein was centrifuged at 20,000 g for 20 mins at 4°C to remove unfolded or aggregated proteins. Concentration of the refolded protein was determined by BCA protein assay (Pierce).

### Expression of ScUlp1 and cleavage of SUMOtFgf15 fusion protein

The procedure for expression and purification of ScUlp1 protease is similar to what has been described above with minor modification to buffers. 10 mM 2-mercaptoethanol was added to all buffers to preserve enzyme activity. ScUlp1 is expressed in *E. coli* cytoplasm in a soluble form, so the supernatant fraction after lysis and centrifugation was used to bind to the Ni-NTA agarose overnight. The binding protein was washed twice the following day and then eluted with 10 ml elution buffer containing 200 mM imidazole. The eluted ScUlp1 protein was dialyzed overnight against 2 L of storage buffer (20 mM Tris-HCl, pH 8.0, 0.3 M NaCl, 10 mM 2-mercaptoethanol) with changing the buffer twice. Glycerol was added to a final concentration of 50% and the purified protein was stored at −80°C. In order to determine the purified ScUlp1 enzyme activity, commercial available SUMO tag protease was compared in parallel, and there was no obvious difference in activity between our protease and the commercial protease (data not shown).

The refolded proteins were incubated with ScUlp1 protease at a 1∶1000 (w/w) ratio for 1 hr at room temperature or overnight at 4°C to remove the N-terminal SUMO tag linked to the tFgf15 (His6-SUMOtFgf15) protein. After centrifugation at 15,000 g, the mixture was passed through Hi-Trap Chelating HP columns (Amersham Biosciences) in buffer (20 mM Tris-HCl, pH 8.0, 0.2 M NaCl) to remove the His6-SUMO tag, and recombinant protein tFgf15 was recovered in the flow-through fractions. Western blot was performed on these samples using a monoclonal anti-His tag antibody (Genescript).

### Activity assay of the recombinant protein tFgf15

Possible residual endotoxin in the recombinant tFgf15 was removed by a Detoxi-Gel Endotoxin Removing Column (Pierce), and the endotoxin concentration in the protein was determined by a GenScript kit. There was no difference between the recombinant protein solution and the control saline solution. Protein concentration was adjusted with saline according to the dose and injection volume of 60 µl per 30 g mouse body-weight. For a dose-dependent experiment, C57BL/6 (n = 3) wild-type (WT) mice, were administered different amounts of tFgf15 protein through tail-vein injection, and their livers were collected 2 hrs after injection. Total RNA from the livers was isolated using the Trizol method (Ambion), and reverse-transcribed to cDNA using MMLV reverse transcriptase (Invitrogen) according to the manufacture's protocol. The mRNA expression of Cyp7a1 was quantified by the Sybr-green-based real-time quantitative-PCR (qPCR) method on the ABI 7900HT system using the primers, mCyp7a1_F: 5′-AACAACCTGCCAGTACTAGATAGC-3′, and mCyp7a1_R: 5′-GTGTAGAGTGAAGTCCTCCTTAGC-3′. The expression of β-actin was determined as an internal normalization control with primers, mbActin_F: 5′-GCGTGACATCAAAGAGAAGC-3′, and mbActin_R: 5′-CTCGTTGCCAATAGTGATGAC-3′.

### Statistical analysis

All experimental data are expressed as the mean ± SE. Multiple groups were tested using one-way ANOVA followed by Dunnett's test to determine which groups were significantly different from the control group. A P value of <0.05 was considered to be statistically significant.

## Results

### Fgf15 signal peptide affected the protein expression in *E. coli*

**Figure 2:**
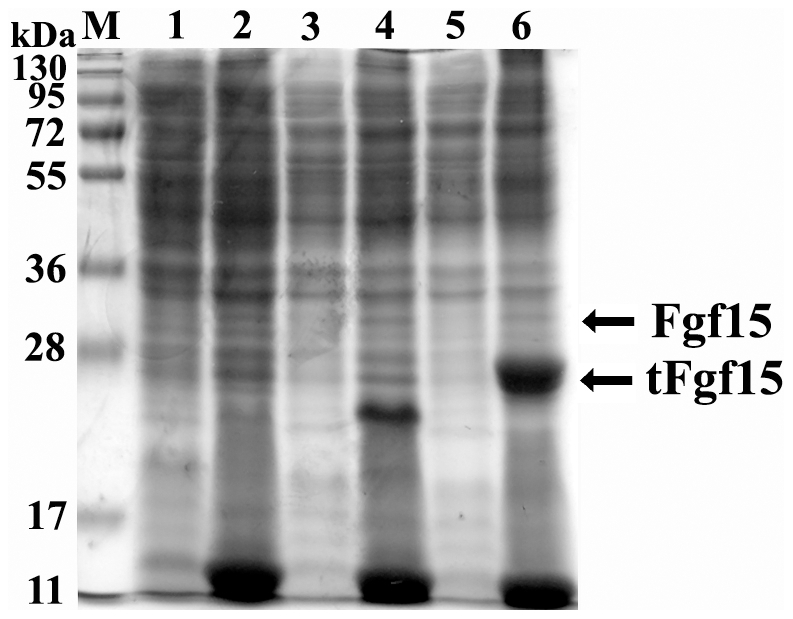
Expression of Fgf15 with or without N-terminal signal peptide in *E. coli*. M: protein molecular weight marker, lane 1: soluble lysate fraction from *E. coli* containing pET28a(+), lane 2: insoluble lysate fraction from *E. coli* containing pET28a(+), lane 3: soluble lysate fraction from *E. coli* containing pET/Fgf15, lane 4: insoluble lysate fraction from *E. coli* containing pET/Fgf15, lane 5: soluble lysate fraction from *E. coli* containing pET/tFgf15, lane 6: insoluble lysate fraction from *E. coli* containing pET/tFgf15.

The full length cDNA of Fgf15, including the predicted N-terminal signal peptide, was cloned from mouse intestine and inserted into a pET28a(+) plasmid to construct pET/Fgf15. Fgf15 contains 218 aa with a predicted 25-aa signal peptide and produces a 25 kDa protein if the full-length protein is expressed. Fgf15 clones were characterized for their expression and solubility in *E. coli* by SDS-PAGE. **Figure 2** shows proteins in lysed cell supernatant and pellet (lanes 3 and 4, respectively). There was no major band at the expected molecular weight in either the supernatant or pellet fraction compared to the control *E. coli* lysate (lanes 1 and 2). A more sensitive western blot analysis was then used to detect Fgf15 expression by His-tag specific antibody, but Fgf15 protein was not detected (data not shown).

The truncated Fgf15 (tFgf15, with molecular weight of 23 kDa) with deletion of the signal peptide was expressed in *E. coli*. The expression and solubility of tFgf15 was determined by SDS-PAGE (**Figure 2**, lanes 5 and 6). Despite high expression levels, there was no protein, at the 23 kDa size, detected in the soluble fraction compared to cell lysate from the empty vector. These results indicate that tFgf15 proteins were expressed at high levels in the form of inclusion bodies. Various conditions were used to optimize culture conditions, including decreasing the incubation temperature (from 25°C to 15°C) and IPTG concentrations (from 1 mM to 0.1 mM). However, Fgf15 protein solubility did not get improved, indicating that Fgf15 protein has a high tendency to aggregate when expressed in *E. coli*. These results demonstrate that the signal peptide in Fgf15 protein may have disturbed the expression of Fgf15 protein in *E. coli*.

### SUMO tag cannot improve the solubility of tFgf15

**Figure 3:**
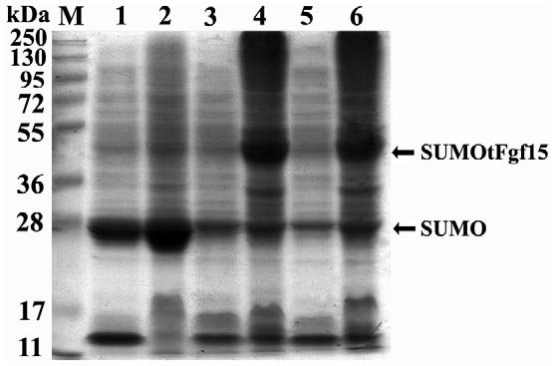
Expression of SUMOtFgf15 in *E. coli*. The solubility of fusion proteins was analyzed on 12% SDS-PAGE gel and stained with Coomassie Brilliant blue. M: protein molecular weight marker, lane 1: total cellular lysate from *E. coli* containing pET/SUMO, lane 2: soluble lysate fraction from *E. coli* containing pET/SUMO, lane 3: insoluble lysate fraction from *E. coli* containing pET/SUMO, lane 4: total cellular lysate from *E. coli* containing pET/SUMOtFgf15, lane 5: soluble lysate fraction from *E. coli* containing pET/SUMOtFgf15, lane 6: insoluble lysate fraction from *E. coli* containing pET/SUMOtFgf15.

The truncated Fgf15 protein was expressed in an insoluble form and was therefore retained in the inclusion bodies. We have performed pilot experiments in an attempt to fuse tFgf15 with GST and Trx, two well-known solubility enhancers, to improve the solubility of Fgf15 protein. However, neither of these tags enhanced Fgf15 protein solubility, even at various culture conditions (data not shown). The SUMO tag has been shown to improve expression levels and solubility, as well as to promote proper folding of many proteins that are difficult to be solubilizied. Therefore, the SUMO tag has been suggested to better enhance solubility than GST, Trx, or MBP tags. We expressed constructs of SUMO and SUMOtFgf15 in *E. coli* (**Figure 3**) and found that most SUMO tags were expressed in the soluble form (lane 2) with less SUMO tag expressed in the insoluble fraction (lane 3). Note that SUMO migrated on the SDS-PAGE gel as more than 20 kDa in size even though it has a molecular mass of 11.5 kDa [18]. **Figure 3** shows that an apparent band of 40 kDa was observed (lane 4, consistent with the calculated molecular mass of recombinant SUMOtFgf15), but there was no obvious SUMOtFgf15 protein expression in the supernatant (lane 5). In addition, all fusion proteins were expressed in the centrifuged pellet fraction, which indicates that all SUMOtFgf15 proteins expressed were insoluble and retained within inclusion bodies. Therefore, the fusion tag SUMO could not improve tFgf15 protein solubility. These results were further confirmed by western blot analysis against the His-tag (data not shown).

Although various highly soluble proteins have been applied as fusion partners to enhance the solubility of recombinant Fgf15 protein, they do not improve solubility when Fgf15 is expressed in *E. coli*. Therefore, *in-vitro* refolding is required to obtain biologically active Fgf15 protein when it is expressed in a prokaryotic system.

### SUMO tag assists in the refolding of Fgf15 *in vitro*

**Figure 4:**
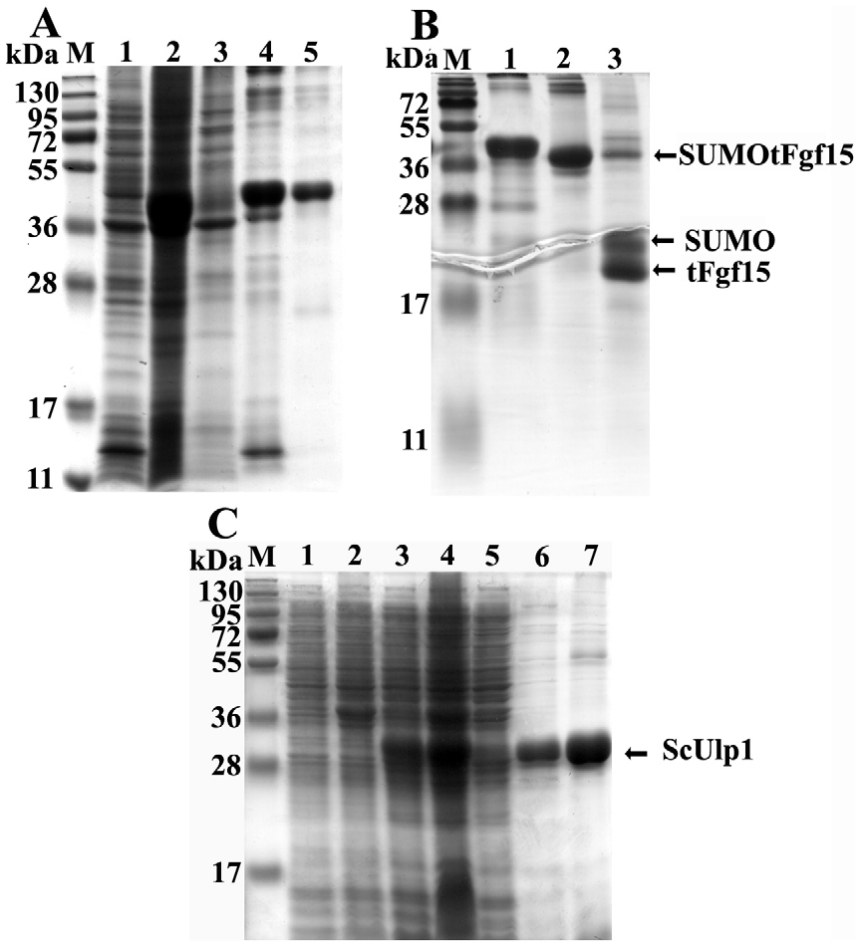
Purification of SUMOtFgf15 inclusion bodies (A), confirmation of SUMOtFgf15 protein refolding following ScUlp1 digestion (B). Panel A lane 1: soluble cell lysate from pET/SUMOtFgf15, lane 2: insoluble inclusion bodies, lane 3: unbound protein after Ni-NTA resin, lane 4: elutes from Ni-NTA by 200 mM imidazole, lane 5: soluble protein after refolding. Panel B lane 1: purified SUMOtFgf15 for starting refolding, lane 2: soluble SUMOtFgf15 protein after refolding, lane 3: refolded SUMOtFgf15 digested by ScUlp1 for 30 mins. Panel C shows the expression and purification of protease ScUlp1. Lane 1–2: lysate from *E. coli* containing pET28a(+) (lane 1, soluble fraction, lane 2: insoluble fraction), lane3–4: lysate from *E. coli* containing pET/ScUlp1 (lane 3: soluble fraction, lane 4: insoluble fraction), lane 5: unbound protein after Ni-NTA resin, lane 6: eluted ScUlp1 by 100 mM imidazole, lane 7: eluted ScUlp1 by 200 mM imidazole.

Fusion proteins of SUMOtFgf15 were extracted from inclusion bodies under denaturing conditions and were purified by Ni-NTA chelating affinity chromatography (Qiagen) before renatured to native state. The purification profile of SUMOtFgf15 is shown in **Figure 4A**. The majority of the fusion protein bound to the Ni-NTA resin, leaving a small amount of the SUMOtFgf15 in solution (lane 3). The SUMOtFgf15 protein was eluted by 200 mM imidazole and purification was efficient as shown by the distinct band in lane 4. After purification, the Fgf15 protein was refolded by stepwise dialysis in the presence of reducing agents to allow for the formation of two native disulfide bridges in the protein. The fusion protein SUMOtFgf15 became soluble after removal of denaturants and reducing reagents by dialysis against PBS buffer (**Figure 4A**, lane 5). Refolding of the purified tFgf15 protein without the SUMO fusion tag was also performed in parallel to compare the effect of SUMO tag on refolding. All tFgf15 protein without SUMO tag precipitated out after removal of denaturing reagent (data not shown). These results suggest that SUMO moiety functions as a chaperone to assist its fusion partners in refolding into correct structure.

The success of refolding was also confirmed by protease ScUlp1 cleavage (**Figure 4B**). ScUlp1 recognizes the tertiary structure of SUMO protein and not just the protease recognition sites [19], [21], which can be used as a method to analyze protein structure. The amino-terminal SUMO moiety and recombinant protein tFgf15 was released from the fusion protein SUMOtFgf15 (**Figure 4B**, lane 3), indicating that at least the SUMO moiety was properly refolded.

The C-terminal of ScUlp1 was expressed at high levels in *E. coli* (**Figure 4C**, lane 3 and 4). Approximately 30% of the total fusion protein expressed was in soluble form. The soluble protein lysate (**Figure 4C**, lane 3) was used for purification of ScUlp1 with Ni-NTA affinity chromatography under reducing conditions. Most soluble proteins were bound to the Ni-NTA resin through the N-terminus His tag. The bound ScUlp1 was eluted by elution buffer containing 100 mM (lane 6) and 200 mM imidazole (lane 7).

### Cleavage of the SUMO tag and purification of the tFgf15 protein

**Figure 5:**
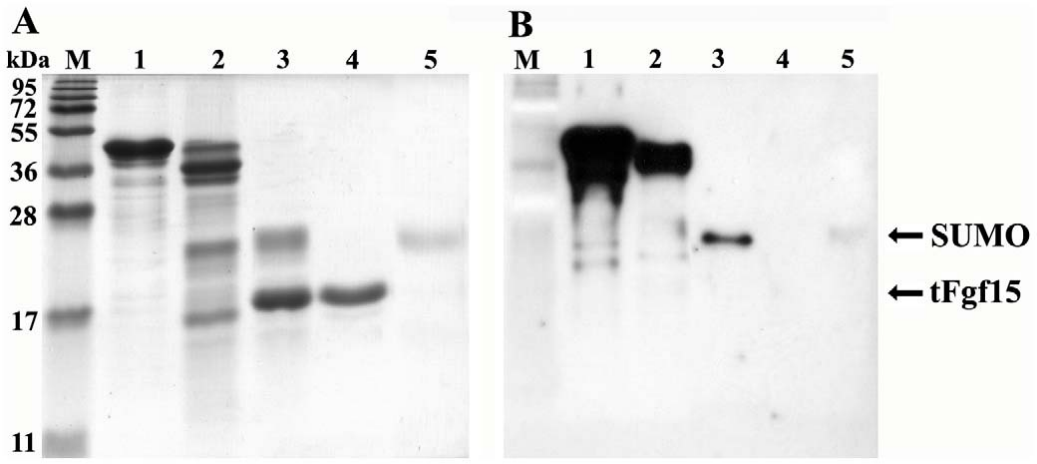
SUMOtFgf15 cleavage and tFgf15 purification by Ni-NTA resin. The samples were separated on 15% SDS-PAGE gel, and stained with Coomassie Brilliant Blue (A) or undergone western blot analysis (B) with anti-His6 tag antibody. M: protein molecular weight marker, lane 1: purified SUMOtFgf15 inclusion bodies, Lane 2: refolded SUMOtFgf15, lane 3: SUMOtFgf15 digested by ScUlp1, lane 4: purified tFgf15 flow through Ni-NTA column, lane 5: eluate from Ni-NTA column using 200 mM imidazle.

Many biological and biomedical applications require protein fusion tag removal from the target protein because the tag may alter the biological activity of the target protein. There is a Gly-Gly motif between SUMO and tFgf15 that can be specifically recognized and cleaved by ScUlp1. **Figure 5A** shows the protein analysis by SDS-PAGE gel after protease cleavage. Fusion protein identity was also assessed by western blot analysis using an antibody against the His tag (**Figure 5B**).

The refolded SUMOtFgf15 protein (lane 2) was effectively cleaved by ScUlp1, and two new bands corresponding to the expected molecular weights, approximately 25 kDa for SUMO and 22 kDa for tFgf15, were detected (lane 3). After applying the cleaved sample to the Ni-NTA column, the N-terminally His6-tagged fusion proteins, including SUMOtFgf15 (non-cleaved), SUMO moiety (cleaved) and protease ScUlp1, bound to the column. The untagged Fgf15 protein did not bind to the column and remained in flow-through solution (lane 4). Coomassie blue staining revealed only one band without any contaminant (**Figure 5A**, lane 4). Therefore the purity of tFgf15 protein was estimated to be greater than 90%. The His-tagged protein that bound to the column was also eluted by imidazole to confirm the identity of the binding proteins (lane 5).

The SUMO tag was removed by ScUlp1 cleavage, which requires reducing buffer conditions containing 2-mercaptoethanol or DTT. However, the reducing regents also broke disulfide bonds in the properly refolded tFgf15 protein. This problem was solved by using an increased amount of ScUlp1 protease for a longer incubation time. The extended overnight incubation without reducing reagents resulted in more than 90% cleavage of the fusion protein (**Figure 5**, lane 3).

Typically, the expression level of SUMOtFgf15 protein in the BL21(DE3) strain was about 10% of the total E. coli protein. Around 200 mg of purified SUMOtFgf15 inclusion bodies could be obtained from 1 liter of bacteria culture media. All proteins were kept soluble after removal of urea through dialysis in the refolding step. However, during the cleavage step, there was some protein precipitation observed by an increase in solution turbidity, which indicates some fusion protein failed to properly refold and thus was precipitated after the SUMO fusion tag was cleaved. At the end of the purification process, we could obtain about 6 mg of purified Fgf15 protein from 1 liter of media, thus the yield of correctly refolded tFgf15 protein from inclusion bodies is about 3%.

### Both SUMO-fused and tFgf15 proteins were biologically active *in vivo*

**Figure 6:**
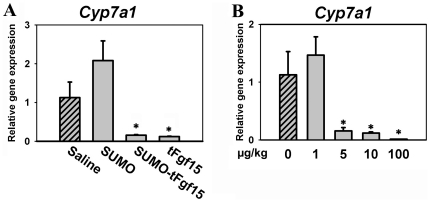
Biological activity of the recombinant Fgf15 proteins. (A) Hepatic Cyp7a1 mRNA levels in mice injected intravenously with saline, fusion protein SUMOtFgf15, or tFgf15 without SUMO fusion tag. (B) Dose-dependency in suppressing hepatic Cyp7a1 gene expression in mice by recombinant tFgf15. * P<0.05, compared to saline-treated group.

Fgf15 is known to reduce the mRNA expression of the Cyp7a1 gene in liver. To assess the biological activity of the recombinant Fgf15 protein *in vivo*, we analyzed the effect of Fgf15 on Cyp7a1 gene expression. Mice were administered SUMOtFgf15 or tFgf15 protein through tail-vein injection. Both SUMOtFgf15 and tFgf15 protein suppressed 90% of hepatic Cyp7a1 mRNA levels, and there was no difference in the extent of suppression between SUMOtFgf15 and tFgf15 protein (**Figure 6A**). In addition, SUMO protein alone did not suppress Cyp7a1 expression, indicating that the biological activity of the SUMOtFgf15 fusion protein comes from the tFgf15 protein and not the SUMO moiety. These results, in combination with those from **Figure 4B**, suggest that both the SUMO moiety and tFgf15 protein have kept their individual structures after refolding *in vitro*. With the assistance of SUMO fusion tag, tFgf15 was properly refolded and maintained its function, and assumedly has kept its tertiary structure after cleavage of the fusion tag.

Biological activity of the recombinant tFgf15 protein was further confirmed in mice (**Figure 6B**). Increasing amounts of recombinant tFgf15 were injected through tail vein into WT mice. Hepatic Cyp7a1 mRNA levels decreased with increasing tFgf15 dosage, indicating that tFgf15 suppresses Cyp7a1 gene transcription in a dose-dependent manner. In detail, as low as 5 µg per kg body weight dosage of recombinant tFgf15 significantly suppressed Cyp7a1 gene expression. These data further indicated that the recombinant tFgf15 protein was biologically active.

## Discussion

The *E. coli* expression system is a fast, inexpensive and widely used system for heteroprotein production. However, this system lacks post-translational modifications, making it an unsuitable system for expressing proteins that require post-translational modifications for their biological activity. Fgf15/FGF19 does not contain potential N-linked glycosylation sites [4], [22], [23], indicating that it does not require post-translational modification, therefore we may use the *E. coli* expression system to express Fgf15 protein *in vitro*. However, there are five cysteines in the Fgf15/FGF19 peptide chain that form two putative disulfide bonds in the protein molecule with one of the two disulfide bonds being conserved throughout the FGF family [4], [22]. It's well known that disulfide bonds are important for maintaining proper protein structure, and thereby protein function. It is also known that the reducing environment in *E. coli* cytoplasm is unfavorable for disulfide bond formation. Previous research on the crystal structure of FGF19 protein[4], [23], which required isolation of the protein from inclusion bodies followed by subsequent in-vitro refolding, suggests that Fgf15, as well as FGF19, may be prone to form inclusion bodies when expressed in *E. coli*. Indeed, the pilot experiments for optimization of *E. coli* culture conditions and fusion expression with various tags such as GST, Trx, and SUMO did not show improvement for increasing Fgf15 protein solubility, indicating that a crucial factor has been missing during Fgf15 refolding, which results in retaining of Fgf15 in the inclusion bodies.

Fgf15 shows a high tendency to be expressed in insoluble form, therefore, *in-vitro* refolding is required to recover bioactive proteins. Protein refolding is not only complicated and time-consuming, but also the mechanism for refolding is still unclear. Therefore, successful refolding of proteins is not guaranteed. It's believed that there are many intermediates formed during the protein refolding process, and refolding and aggregation appear to occur in parallel. Nonetheless aggregation-prone intermediates form early in the protein folding process. Therefore, proper protein refolding depends on a competition between correct folding and aggregation. In many cases, formation of the correct disulfide bond is a rate-limiting step during the refolding process and incorrect disulfide bond formation leads to protein precipitation. To prevent aggregation, many small chemical compounds, such as L-arginine, have been used successfully to prevent protein aggregation and to enhance correct protein folding [9]. However, there is no report of protein fusion tags for enhancing *in-vitro* refolding.

SUMO protein has been shown to markedly enhance the expression and solubility of its fusion proteins, and the mechanism in part lies in the structure of SUMO protein which contains an external hydrophilic surface and inner hydrophobic core [18], [19]. This structure may exert a detergent-like effect on its linked proteins, which may possess robust folding characteristics, resulting in refolding of SUMO faster in the fusion protein during the refolding process. Folded SUMO may function as a general molecular chaperone to prevent aggregation of intermediates, therefore keeping folding intermediates in solution long enough to adopt correct conformations [9], [10], [24], [25]. According to this theory, a two-step dialysis was used to refold the SUMOtFgf15 protein. First, denaturing reagents were removed stepwise to make the protein form a tight conformation in the presence of reducing reagents that were used to prevent formation of improper disulfide bonds. Second, oxidation was introduced to form disulfide bonds to maintain the correct structure of proteins. Even though the mechanism is not elucidated, it is clear that SUMO tag assists the fused Fgf15 protein to recover from misfolding inclusion bodies. The recovered proteins have strong biological activities, indicating that Fgf15 has gained proper conformation after refolding.

It is well known that inclusion bodies can simplify the protein isolation process. However, proper protein refolding after inclusion body isolation is a great challenge [9], [10]. We have demonstrated that SUMO is a potential solution to this problem because it promotes *in-vitro* refolding by helping its partner protein to quickly fold in its correct conformation. In addition, SUMO has solubility-enhancing characteristics. In conclusion, the use of fusion partner SUMO is an effective system for heterologous protein expression. In combination with the easy isolation and purification of inclusion bodies, SUMO offers the advantage of effective *in-vitro* refolding of its partner proteins and can greatly accelerate the recombinant protein production.

## References

[1] Inagaki T, Choi M, Moschetta A, Peng L, Cummins CL (2005). Fibroblast growth factor 15 functions as an enterohepatic signal to regulate bile acid homeostasis.. Cell Metab.

[2] Xie MH, Holcomb I, Deuel B, Dowd P, Huang A (1999). FGF-19, a novel fibroblast growth factor with unique specificity for FGFR4.. Cytokine.

[3] Chiang JY (2009). Bile acids: regulation of synthesis.. J Lipid Res.

[4] Goetz R, Beenken A, Ibrahimi OA, Kalinina J, Olsen SK (2007). Molecular insights into the klotho-dependent, endocrine mode of action of fibroblast growth factor 19 subfamily members.. Mol Cell Biol.

[5] Tomlinson E, Fu L, John L, Hultgren B, Huang X (2002). Transgenic mice expressing human fibroblast growth factor-19 display increased metabolic rate and decreased adiposity.. Endocrinology.

[6] Nicholes K, Guillet S, Tomlinson E, Hillan K, Wright B (2002). A mouse model of hepatocellular carcinoma: ectopic expression of fibroblast growth factor 19 in skeletal muscle of transgenic mice.. Am J Pathol.

[7] Fu L, John LM, Adams SH, Yu XX, Tomlinson E (2004). Fibroblast growth factor 19 increases metabolic rate and reverses dietary and leptin-deficient diabetes.. Endocrinology.

[8] Baneyx F (1999). Recombinant protein expression in Escherichia coli.. Curr Opin Biotechnol.

[9] Mayer M, Buchner J (2004). Refolding of inclusion body proteins.. Methods Mol Med.

[10] Burgess RR (2009). Refolding solubilized inclusion body proteins.. Methods Enzymol.

[11] Makrides SC (1996). Strategies for achieving high-level expression of genes in Escherichia coli.. Microbiol Rev.

[12] Kapust RB, Waugh DS (1999). Escherichia coli maltose-binding protein is uncommonly effective at promoting the solubility of polypeptides to which it is fused.. Protein Sci.

[13] Smith DB, Johnson KS (1988). Single-step purification of polypeptides expressed in Escherichia coli as fusions with glutathione S-transferase.. Gene.

[14] Bedouelle H, Duplay P (1988). Production in Escherichia coli and one-step purification of bifunctional hybrid proteins which bind maltose. Export of the Klenow polymerase into the periplasmic space.. Eur J Biochem.

[15] di Guan C, Li P, Riggs PD, Inouye H (1988). Vectors that facilitate the expression and purification of foreign peptides in Escherichia coli by fusion to maltose-binding protein.. Gene.

[16] Davis GD, Elisee C, Newham DM, Harrison RG (1999). New fusion protein systems designed to give soluble expression in Escherichia coli.. Biotechnol Bioeng.

[17] LaVallie ER, DiBlasio EA, Kovacic S, Grant KL, Schendel PF (1993). A thioredoxin gene fusion expression system that circumvents inclusion body formation in the E. coli cytoplasm.. Biotechnology (N Y).

[18] Butt TR, Edavettal SC, Hall JP, Mattern MR (2005). SUMO fusion technology for difficult-to-express proteins.. Protein Expr Purif.

[19] Malakhov MP, Mattern MR, Malakhova OA, Drinker M, Weeks SD (2004). SUMO fusions and SUMO-specific protease for efficient expression and purification of proteins.. J Struct Funct Genomics.

[20] Hay RT (2005). SUMO: a history of modification.. Mol Cell.

[21] Hay RT (2007). SUMO-specific proteases: a twist in the tail.. Trends Cell Biol.

[22] Nishimura T, Utsunomiya Y, Hoshikawa M, Ohuchi H, Itoh N (1999). Structure and expression of a novel human FGF, FGF-19, expressed in the fetal brain.. Biochim Biophys Acta.

[23] Harmer NJ, Pellegrini L, Chirgadze D, Fernandez-Recio J, Blundell TL (2004). The crystal structure of fibroblast growth factor (FGF) 19 reveals novel features of the FGF family and offers a structural basis for its unusual receptor affinity.. Biochemistry.

[24] Jungbauer A, Kaar W (2007). Current status of technical protein refolding.. J Biotechnol.

[25] Wingfield PT, Palmer I, Liang SM (2001). Folding and purification of insoluble (inclusion body) proteins from Escherichia coli.. Curr Protoc Protein Sci Chapter.

